# The murine vaginal microbiota and its perturbation by the human pathogen group B *Streptococcus*

**DOI:** 10.1186/s12866-018-1341-2

**Published:** 2018-11-26

**Authors:** Alison Vrbanac, Angelica M. Riestra, Alison Coady, Rob Knight, Victor Nizet, Kathryn A. Patras

**Affiliations:** 1 Division of Host-Microbe Systems and Therapeutics, Department of Pediatrics, University of California San Diego, 9500 Gilman Dr, MC 0760, La Jolla, CA 92093-0760 USA; 2 Center for Microbiome Innovation, University of California San Diego, La Jolla, CA USA; 3 Department of Computer Science and Engineering, University of California San Diego, La Jolla, CA USA; 4 Skaggs School of Pharmacy and Pharmaceutical Sciences, University of California, San Diego, La Jolla, CA USA

**Keywords:** Vaginal microbiome, Murine model, Estrous cycle, Group B *Streptococcus*, 16S rRNA sequencing

## Abstract

**Background:**

Composition of the vaginal microbiota has significant influence on female urogenital health and control of infectious disease. Murine models are widely utilized to characterize host-pathogen interactions within the vaginal tract, however, the composition of endogenous vaginal flora remains largely undefined with modern microbiome analyses. Here, we employ 16S rRNA amplicon sequencing to establish the native microbial composition of the vaginal tract in adult C57Bl/6 J mice. We further interrogate the impact of estrous cycle and introduction of the human vaginal pathobiont, group B *Streptococcus* (GBS) on community state type and stability, and conversely, the impact of the vaginal microbiota on GBS persistence.

**Results:**

Sequencing analysis revealed five distinctive community states of the vaginal microbiota dominated largely by *Staphylococcus* and/or *Enterococcus*, *Lactobacillus*, or a mixed population. Stage of estrus did not impact microbial composition. Introduction of GBS decreased community stability at early timepoints; and in some mice, GBS became the dominant bacterium by day 21. Endogenous *Staphylococcus* abundance correlated with GBS ascension into the uterus, and increased community stability in GBS-challenged mice.

**Conclusions:**

The murine vaginal flora is diverse and fluctuates independently of the estrous cycle. Endogenous flora may impact pathogen colonization and dissemination and should be considered in urogenital infection models.

**Electronic supplementary material:**

The online version of this article (10.1186/s12866-018-1341-2) contains supplementary material, which is available to authorized users.

## Background

The vaginal microbiota is intimately linked to women’s health. In humans, the vaginal microbiota exists in 5 distinct community state types (CSTs) which are generally dominated by *Lactobacillus* spp. [1]. Composition of the microbiota varies in temporal stability, with greatest instability exhibited during menses [2]. Subcategory CST IV-A, a community dominated by facultative and strict anaerobes in place of *Lactobacillus* spp. [1], has been associated with increased incidence of vulvovaginal atrophy [3], and colonization by group B *Streptococcus* (GBS) [4]. Furthermore, vaginal dysbiosis, denoted clinically as bacterial vaginosis (BV), is characterized as a heterogeneous vaginal microbiota not dominated by a single taxon [5]. BV or high bacterial diversity has been associated with adverse health outcomes including preterm birth [6, 7], HIV acquisition [8, 9], and infection with other urogenital pathogens including *Trichomonas vaginalis* [10, 11], *Chlamydia trachomatis* [12], and *Neisseria gonorrhoeae* [13]. The vaginal microbiota represents a key constituent of host-pathogen interactions in the vaginal mucosa, with significant implications for understanding susceptibility to disease and optimizing prevention and treatment strategies.

Murine models of vaginal infection and colonization are commonly used to characterize microbial pathogenesis determinants, host immune responses, and therapeutic interventions for urogenital pathogens including HIV [14], group B *Streptococcus* [15], *Candida albicans* [16, 17], *Trichomonas vaginalis* [18], *Gardnerella vaginalis* [19], and *Chlamydia trachomatis* [20]. Despite the widespread use of this animal model, the commensal murine vaginal microbiota has yet to be longitudinally characterized with modern microbiome sequencing methods. Earlier culture-based studies identified *Enterobacteriaceae*, *Streptococcus* spp., *Staphylococcus* spp., *Corynebacterium* spp., and *Lactobacillus* spp. among the murine vaginal flora [21, 22], while 16S sequence-based studies have either focused on select microbes of interest [23] or have failed to account for estrus [24]. Variation in vaginal communities across time and in relation to estrus have not been described.

Integrating detailed knowledge of the mouse vaginal microbiota into host-pathogen infections can provide a more direct application for these models to human urogenital pathogens. In this study, we characterize the murine vaginal microbiota of the post-pubertal female C57Bl/6 J Jackson mouse over time to assess the composition and stability of the vaginal flora throughout the estrous cycle. We also introduce the vaginal commensal bacterium and opportunistic pathogen, GBS to evaluate microbiota changes in the context of a relevant human infection model.

## Results

### Murine vaginal microbiota can be categorized into distinct community state types

Little is known about the compositional stability of the murine vaginal flora. Mice have short estrous cycles that last 4–5 days and consist of four stages: proestrus, estrus, metestrus, and diestrus [25].To assess stability of the mouse vaginal microbiota in this context, we used one of the most commonly utilized mouse strains/ages and sources: post-pubertal 8-week-old female C57Bl/6 J mice, obtained from Jackson Laboratories. Mice were received at 7 weeks of age, randomized upon arrival into 5 mice per cage, and acclimated over a one-week period. We longitudinally sampled the vaginal microbiota every three days over a period of 15 days by lavaging with phosphate-buffered saline (PBS). We lavaged a second set of mice only twice, on days 0 and 15, to test whether frequent lavaging itself alters the microbiota. The corresponding estrous cycle stage of each lavage sample was determined by light microscopy. Samples were processed for 16S rRNA gene sequencing as detailed in the Methods section. Contaminants from sequencing reagents (primarily *Pseudomonas*, *Geobacillus*, and *Sphingobium* reads) or chloroplast and mitochondrial sequences were removed before assessing community composition.

**Fig. 1:**
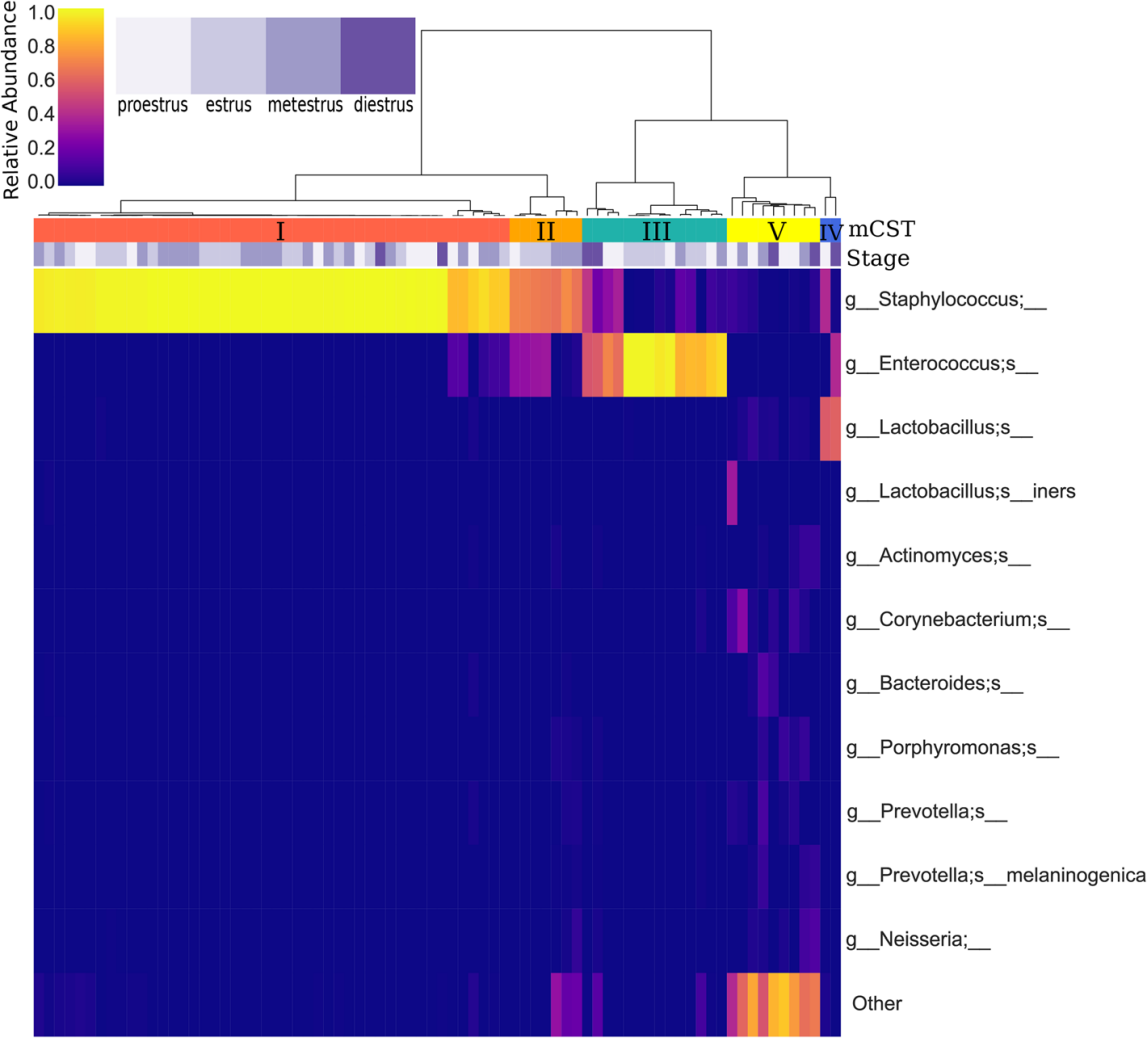
Murine community state types and bacterial landscape of the murine vaginal microbiota. Samples are clustered by community state with Ward’s linkage of Euclidean distances (silhouette score of 0.732, sklearn). Estrous cycle stage is depicted by shades of purple and bacterial abundances are indicated by heatmap intensity corresponding to the colorbar ranging from purple to yellow

For comparison to earlier human vaginal microbiome characterization studies [1, 2], samples were assigned murine community state types (mCST) by hierarchical clustering with Ward’s linkage of Euclidean distances (Fig. 1) (silhouette score of 0.732), with the rarefied OTU table at 1500 reads per sample. The most predominant community state type, mCST I, consisted of *Staphylococcus*-dominant flora. mCST II samples contained vaginal flora comprised primarily of both *Staphylococcus* and *Enterococcus*, while mCST III was predominantly *Enterococcus*. mCST IV samples were *Lactobacillus*-dominant and mCST V samples were not dominated by either *Staphylococcus* or *Enterococcus* and had higher alpha diversity (*p* < 0.001), Shannon diversity and observed operational taxonomic units (OTU). Additionally, there was one single sample dominated by *Bifidobacterium*, and this sample was excluded from subsequent analyses as it was thought to be contaminated.

### Murine vaginal community states are unstable

**Fig. 2:**
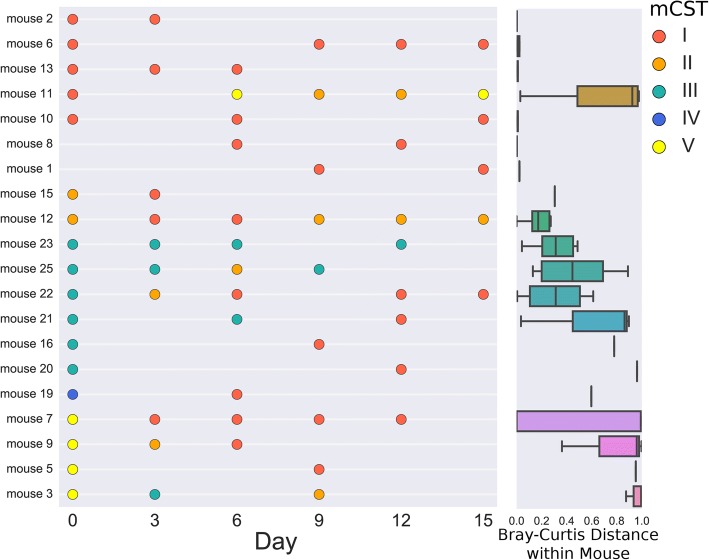
Vaginal Microbiome Stability. Mice from the cohort sampled every three days are displayed ordered by mouse and time point (left). Missing samples indicate sequencing failure (< 1500 reads/sample) and point color depicts the community state type of the sample. Mice with fewer than two successfully sequenced samples were excluded. Bray-Curtis distances between all samples from each individual mouse (right)

Interestingly, community states were relatively unstable in mice. Of mice that had at least two successfully sequenced samples (at least 1500 reads/sample), 70% had samples in at least two different community states (Fig. 2). mCST I appeared to be the most stable community state: for samples from consecutive timepoints that successfully sequenced, 12/14 mCST I samples were assigned mCST I at the next time point (Fig. 2). In comparison, only 1/5 samples in mCST II were also mCST II at the next consecutive time point.

### Estrous cycle does not impact vaginal community state type

**Fig. 3:**
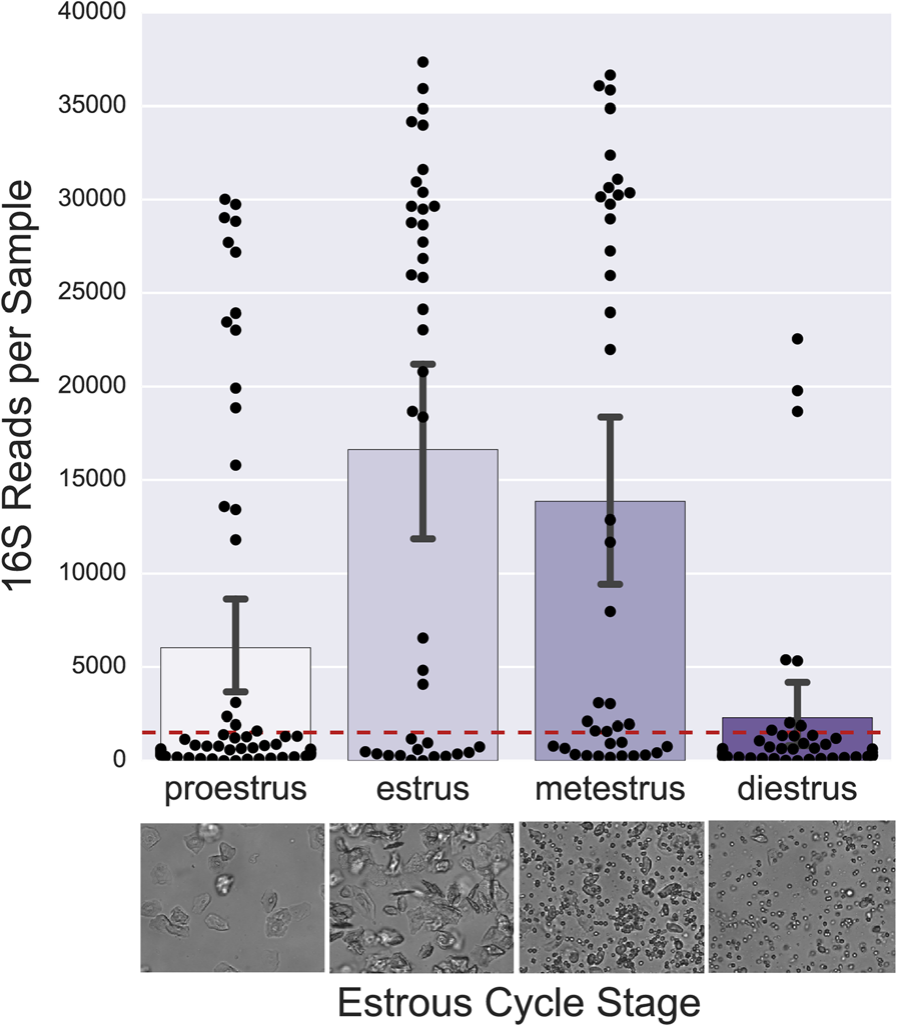
Estrous cycle stage influences 16S rRNA sequencing success. 16S rRNA sequences after removing contaminants, mitochondria, and chloroplast sequences (top). Images (bottom) depict representative microscopy images from vaginal lavages in the different estrous cycle stages, Magnification: 200X

**Fig. 4:**
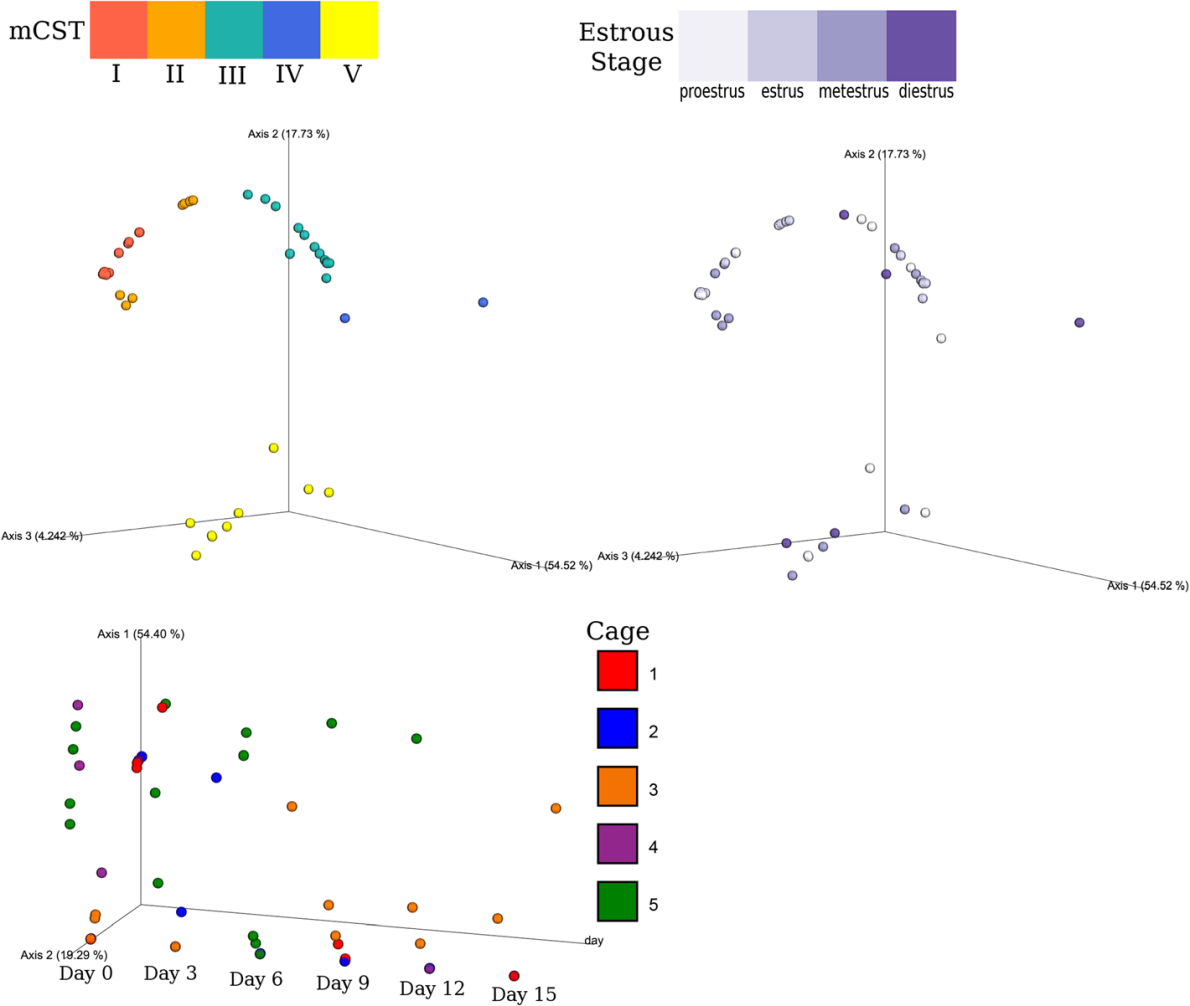
Significant clustering by mCST, but not estrous stage. PCoA Plots of the Bray-Curtis distance matrix of mCST (top left), estrous stage (top right), and cage (bottom). Clustering by mCST was significant (*p* = 0.001, pseudo-F-statistic = 52.13, by PERMANOVA with 999 permutations), while clustering by estrous stage was not (*p* = 0.089, pseudo-F statistic = 1.69, by PERMANOVA 999 permutations). Clustering by cage was only significant on days 0 (*p* = 0.002, pseudo-F-statistic = 3.00) and 9 (*p* = 0.001, pseudo-F-statistic = 35.5)

After filtering and rarefaction to 1500 reads per sample, few samples staged as diestrus and proestrus remained (19.5 and 31.1% respectively) compared to estrus and metestrus (64.1, and 66.6% respectively, Fig. 3). Though sequencing success may vary over estrous cycle stage, community state type was not significantly associated with different stages in the estrous cycle (X^2^ = 17.29, *p* = 0.138). A random forest classifier (scikit-learn) for estrous cycle stage performed on the rarefied OTU table achieved an accuracy of only 0.125, indicating that bacterial composition is a poor predictor of estrous cycle stage. Additionally, beta diversity clustering for estrous cycle stage was not significant and clustering by cage, stratified by day, was only significant on days 0 and 9. (Fig. 4).

### Group B *Streptococcus* challenge destabilizes vaginal community states

To investigate how urogenital pathogens may perturb the murine vaginal microbiota, we longitudinally sampled a second cohort of mice experimentally challenged with GBS and compared them to an uninfected control group. These mice received intraperitoneal injection of 0.5 mg β-estradiol 24 h before infection to synchronize estrus and promote colonization [26]. Mice were lavaged and vaginally inoculated with 1 × 10^7^ colony forming units (CFU) of a well characterized human serotype III GBS isolate COH1 in 10 μl PBS or 10 μl PBS alone (control), then lavaged 3, 7, 14, and 21 days after infection for microbiome sampling. GBS CFUs were also monitored by plating. On days 3, 14, and 21, twelve mice per group were sacrificed to determine bacterial tissue burdens. Lavage samples were processed for 16S rRNA sequencing following the same protocol as the staging cohort.

Although these mice were ordered in a separate shipment, they exhibited similar vaginal microbiota and community-state type clustering (Additional file 1). Community state type mCST I consisted of *Staphylococcus*-dominant flora and mCST II samples contained vaginal flora primarily comprised of *Staphylococcus* and *Enterococcus*, and in some cases, *Streptococcus* reads from GBS colonization. mCST VI samples were dominated by GBS (*Streptococcus*), while mCST III was predominantly *Enterococcus*. *Lactobacillus* comprised most of the flora in mCST IV samples and mCST V samples consisted of a mix of bacteria. *Streptococcus* 16S reads were only abundant in GBS-colonized mice and deblurred 16S sequences mapped to *Streptococcus agalactiae* (GBS) in Genbank. To preserve more samples for longitudinal analysis, samples were rarefied to 500 reads per sample (rarefaction plots in Additional file 2).

**Fig. 5:**
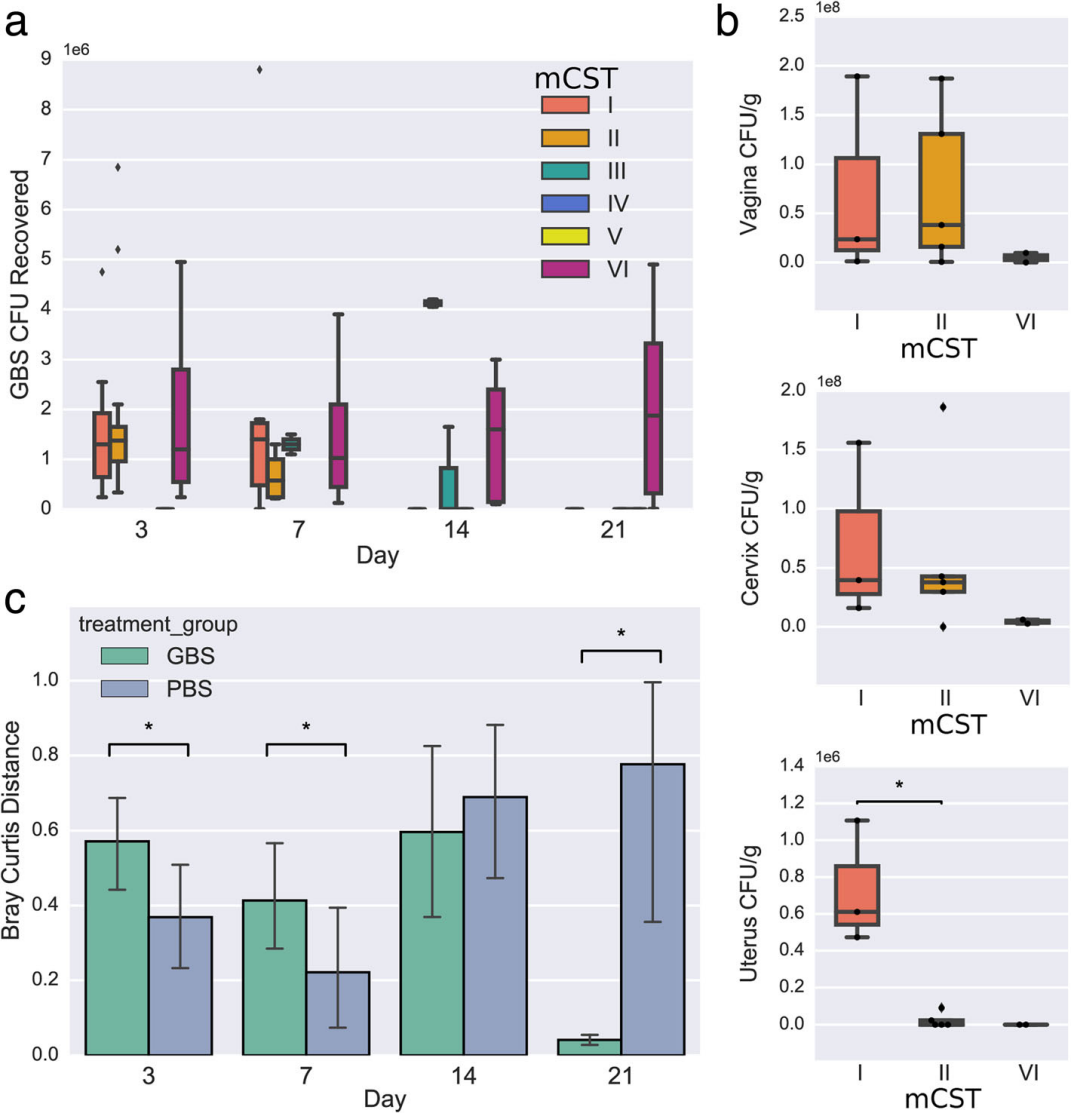
GBS colonization across community states. **a** GBS CFU recovered from vaginal lavage fluid and grouped according to mCST classification on day 0. **b** GBS CFU recovered from tissues on day 3 and grouped according to mCST classification on day 0 **c** Bray-Curtis distance of vaginal lavage sequences of GBS and PBS groups over time. Statistical significance indicated with asterisk (*p* < 0.05, Mann-Whitney U Test)

**Fig. 6:**
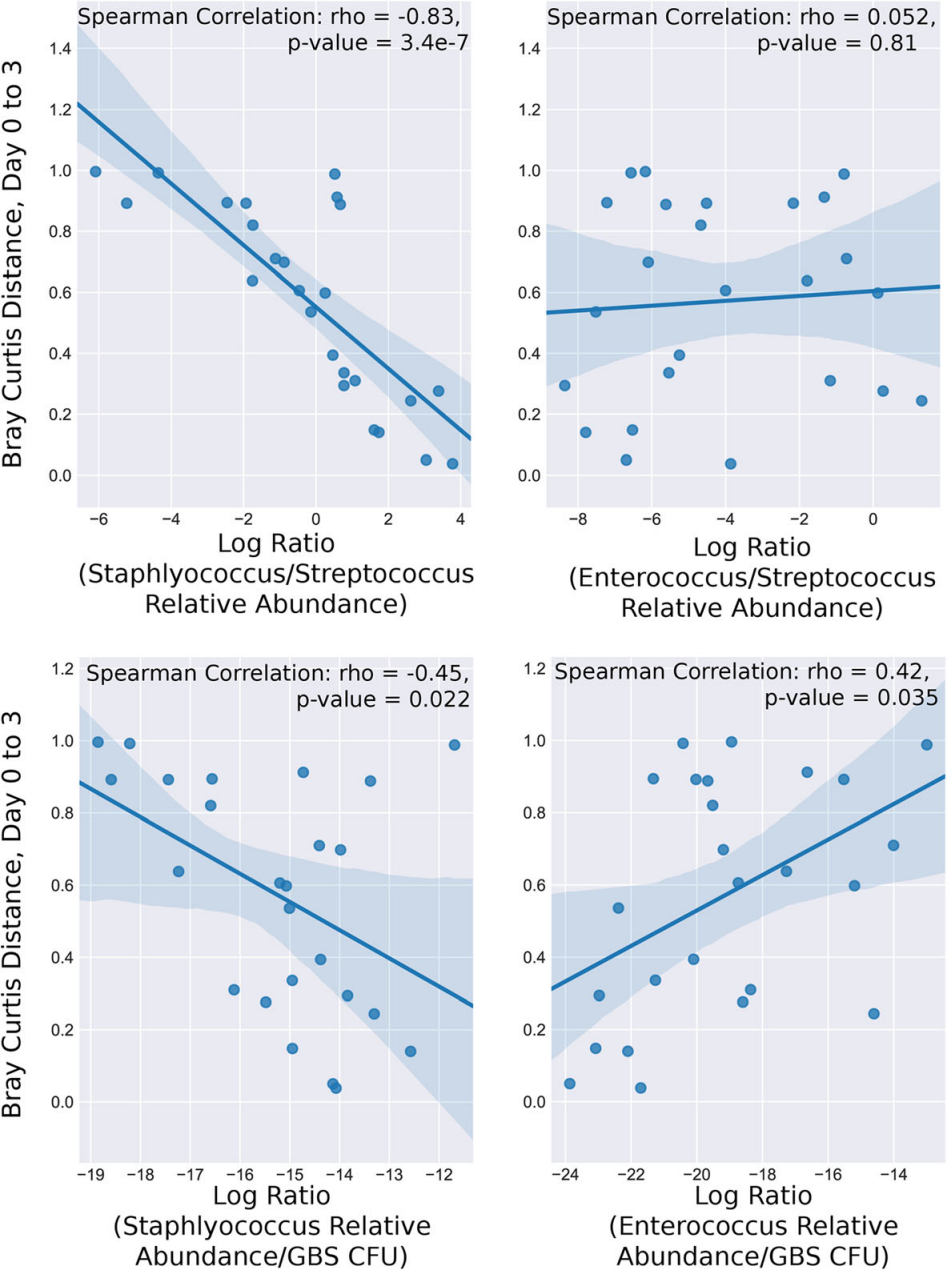
Community instability induced by GBS colonization correlates with *Staphylococcus* GBS turnover. Spearman correlations of pairwise Bray-Curtis distance between day 0 and day 3 samples with the log ratio of *Staphylococcus* relative abundance over *Streptococcus* relative abundance (top left) or GBS CFU (bottom left) and *Enterococcus* relative abundance over *Streptococcus* relative abundance (top right) or GBS CFU (bottom right)

Though mCST VI represented the GBS-dominant state, GBS CFU determined by plating of vaginal lavage samples was not significantly different at day 3 or 7 across all mCSTs (ANOVA *p* = 0.68, *p* = 0.75, respectively)(Fig. 5a). However, by day 21 GBS CFU were only detected in mice with mCST VI where GBS had completely overtaken the vaginal flora (Fig. 5a). Initially, GBS challenge significantly destabilized the vaginal microbiota; pairwise Bray-Curtis distances between consecutive days for individual mice revealed significantly increased distances in the GBS-challenged mice for days 3 (distance from day 0 to day 3) and 7 (distance between days 3 and 7) (Fig. 5c). By day 21, the majority of the GBS mice had GBS-dominant flora (mCST VI), significantly reducing the pairwise distance between days 14 and 21 compared to PBS mice. To look specifically at how changes in prominent taxa were contributing to the initial increased pairwise sample distance with GBS colonization while minimizing the effects of compositionality, we took the log ratio of *Staphylococcus* relative abundance over *Streptococcus* relative abundance or GBS CFU and *Enterococcus* relative abundance over *Streptococcus* relative abundance or GBS CFU. Correlating these log ratios to the Bray-Curtis distances between the day 0 and day 3 samples within mice revealed that a decrease in the ratio of *Staphylococcus* to *Streptococcus* or GBS CFU was significantly correlated with increased distance between samples. Conversely, the ratio of *Enterococcus* to *Streptococcus* had no significant correlation with Bray-Curtis distance and the log ratio of *Enterococcus* relative abundance to GBS CFU was correlated with reduced distance between samples (Fig. 6). As the ratio of *Staphylococcus* to GBS is associated with community instability (distance), this suggests that turnover of *Staphylococcus* for GBS is contributing to the increase in pairwise distances.

For GBS-challenged mice, only 8/27 samples assigned to mCST I remained mCST I at the next consecutive time point. In comparison, control PBS mice had 19/27 mCST I samples assigned mCST I at the next consecutive time point. Additionally, mCST I mice exhibited significantly higher CFU/g in the uterus on day 3 (Fig. 5b) and uterus CFU was significantly correlated with *Staphylococcus* relative abundance (spearman, *p* = 0.0027), but this difference was nullified at later time points. By day 21, only mCST VI mice had GBS CFU remaining in vaginal, cervical, and uterine tissue **(**Additional file 3).

## Discussion

Like humans, laboratory mouse strain C57Bl/6 J exhibit distinct vaginal microbiota community states (here named mCSTs) generally dominated by single bacterial taxa (Fig. 1). Three of the murine vaginal microbiota mCSTs were dominated by bacteria from different genera of Gram-positive facultative anaerobes: *Staphylococcus*, *Enterococcus*, and *Lactobacillus.* This dramatic variation in vaginal flora composition within an in-bred strain of mice raises the question of whether the commensal microbiota should be monitored in murine vaginal colonization and infection models. It is also likely that vaginal microbiota varies across vivaria, vendor, and mouse strain. Previous studies have examined the vaginal microbiota of BALB/c [24] and ICR mice [21]. In the ICR study, culture-based techniques revealed frequent presence of *Streptococcus/Enterococcus*, *Staphylococcus*, *Lactobacillus,* and Gram-negative rods [21]. In the BALB/c study, 16S amplicon sequencing described the most abundant phyla were *Firmicutes* and *Proteobacteria*, with distinctive separation of vaginal communities into two subclusters, one of which was dominated by *Streptococcus* [24]. Although the consistent presence of organisms such as *Staphylococcus*, *Enterococcus*, and *Lactobacillus* suggest there may be a core set of vaginal organisms across mouse strains, direct comparison of our results with these studies is difficult due to differences in sampling and analyses and smaller sample size (10 BALB/c mice and 27 ICR mice). Our findings indicate that the urogenital pathogens under study may encounter and interact with completely different commensal vaginal flora, even within the same cage of mice, potentially introducing variation and influencing experimental outcomes.

Numerous studies have examined constituents controlling vaginal persistence in mice including innate and adaptive immune responses [27–29] and GBS regulatory and virulence factors [30–32]. Additionally, several studies have noted GBS in the vaginal lumen in close proximity to native vaginal flora [29, 33]; however, none to date have examined the role of native vaginal microbiota in this model. Our study did not establish a definitive role for the microbiota in experimental GBS challenge, and this may be due to the high inoculum dose utilized in this model, which typically achieves > 90% colonization within the first week post-infection [26]. Future studies should examine a titration of inoculum to reveal more subtle contributions of the endogenous flora on GBS persistence. Nonetheless, we did note the otherwise relatively stable *Staphylococcus*-dominated CST I was perturbed by GBS challenge. Mice with mCST I also exhibited increased GBS ascension into uterine tissue by day 3. In multiple human studies, GBS has been co-isolated with *Staphylococcus* spp. in both pregnant and non-pregnant women [4, 34, 35]. Additionally, in invasive GBS polymicrobial infections, *S. aureus* is the most frequently co-isolated organism [36, 37]. One study demonstrated induction of *S. aureus* toxic shock syndrome toxin-1 by GBS culture supernatants in vitro [38], but whether this phenomenon occurs in the vaginal mucosa, or whether GBS virulence is also impacted, remains to be described.

In humans, vaginal CST does not generally correlate with GBS colonization status, except for the sub-group CST IV-A, a non-*Lactobacillus* dominant state [4]. Antagonism between *Lactobacillus* and GBS has been reported in both in vitro [39–41] and in vivo [42] model systems. Oral probiotics containing *Lactobacillus* have demonstrated efficacy in controlling GBS colonization in pilot human trials [43, 44]. Likewise, in mice dominated by *Lactobacillus* (CST IV), we were unable to detect any GBS beyond day 7, however, our sample size was too small (*n* = 2) to observed statistical differences. Furthermore, we observed that GBS was able to completely overtake the vaginal microbiota in some mice (> 80% of 16S reads), even 21 days post-infection. These mice had significantly higher GBS CFU over the course of the study than mice with flora that was not taken over by GBS on day 21 (student’s t-test, t-stat = 2.45, *p* = 0.018).

To our knowledge, the acquisition and maturation of the murine vaginal microbiota has not yet been examined. In this study, we used post-pubertal, non-pregnant mice, 8 weeks of age, which is a common age used to model urogenital diseases [17, 26]. At this age, the estrous cycle does not appear to influence the composition of the vaginal microbiota, but does impact sequencing success (Fig. 3). In line with this observation, previous studies using culture-based quantification have reported increased bacterial abundance in estrus compared to diestrus [21, 27, 45]. We selected vaginal lavage as our sampling method due to precedence in the literature for > 90% bacterial recovery [45, 46] and for inter-sample consistency and collection volume between our cohorts. To overcome the low biomass resulting from vaginal lavage, future studies may consider alternatives such as swabbing or scraping to obtain more biomass from the mucosa. Though physiology and immune cell populations fluctuate throughout the estrous cycle, potential variability in bacterial abundance provides further impetus for synchronization. The precedence for steroid hormone administration to achieve optimal infection of urogenital pathogens in murine models supports this observation [47, 48].

While factors such as mouse strain, facility, and vendor could certainly influence mCSTs as observed with gut microbiota [49, 50], our two separate studies and shipments of mice exhibited remarkable vaginal mCST homogeneity (Additional file 1) with the exception of an emerging *Streptococcus*-dominated mCST in GBS-infected mice. Future microbiome sequencing of the murine vaginal microbiota extended to other ages, strains, vendors, and vivaria, may reveal the existence of more community states or sub-groups within mCSTs.

## Conclusions

This current study is the first to our knowledge to characterize the murine vaginal microbiota throughout estrus using 16S rRNA sequencing. We further demonstrate the influence of endogenous flora on successful colonization and by a human pathogen. This work underscores the importance of continuing to assess the native murine flora in models of human vaginal pathogens.

## Methods

### Mouse model and sample collection

All animal studies were reviewed and approved by the UC San Diego Animal Care and Use Committee and conducted using accepted veterinary standards. Mice were maintained on a 12 h light/dark cycle, with controlled temperature (19–22 °C) and 40–60% humidity. Mice were fed a commercial diet (2020X, Teklad) and sterile water ad libitum. See Additional file 4 for an overview of experimental design. For the estrous staging study (Study 1), 7-week-old female C57Bl/6 J mice (*n* = 40) were purchased from the Jackson Laboratory and housed five mice per cage. Mice were allowed to acclimate for one week prior to sample collection. To sample the vaginal microbiota, mice were manually restrained, and lavaged twice with 50 μl of sterile phosphate-buffered saline (PBS) using 200 μl Gel-Loading pipet tips (Fisher Scientific) in a laminar flow hood [26]. To control for environmental contamination, each day that lavage was performed, we used a single container of PBS, open in the hood during the entire sampling time. Every pipette tip was introduced to the same PBS container before lavaging. At the end of sample collection, an aliquot of this PBS was collected and used as a control for sequencing contamination. Twenty-five mice were lavaged every 3 days for 15 days, and fifteen mice were lavaged only twice, 15 days apart. After collecting lavage samples, 3 μl was removed from each sample for estrous cycle staging. Samples were visualized with a Zeiss Observer.D1 microscope at 200X magnification and the estrous cycle stage was identified independently by two individuals with results corroborated. For the GBS pathogen challenge study (Study 2), 7-week-old female C57Bl/6 mice (*n* = 72) were purchased from the Jackson Laboratory, housed four mice per cage, and allowed to acclimate for 1 week before infection. For GBS inoculation, we utilized a previously described GBS vaginal colonization model [26]. Briefly, mice were first injected IP with 0.5 mg of beta-estradiol suspended in 100 μl sesame oil, and 24 h later, mice were vaginally inoculated with 1 × 10^7^ CFU of GBS COH1 [51] in 10 μl of PBS or PBS only as a control. Vaginal lavage sampling as described above was performed on days 0, 3, 7, 14, and 21 post-inoculation. For tissue collection at days 3, 14, and 21, mice were sacrificed and vagina, cervix, and uterus dissected and homogenized in PBS using 1 mm silica beads (Biospec) and shaken at 6000 rpm for 60 s using a MagNA Lyser (Roche). To quantify bacterial burdens, tissue homogenates were diluted and plated on CHROMagar StrepB (DRG International) to distinguish GBS CFU as light pink or mauve colonies.

### 16S rRNA gene amplicon sequencing

Sample processing was performed following the Earth Microbiome Project [52] DNA extraction and 16S sequencing protocol, detailed on the EMP website: http://www.earthmicrobiome.org/protocols-and-standards/16s/. In brief, lavage sample DNA was extracted using the 96-well MoBio Powersoil DNA kit. Barcoded 515F-806R primers targeting the V4 region of the 16S rRNA gene were used to for 16S amplification, and the resulting V4 amplicons were sequenced at UCSD Institute for Genomic Medicine (IGM) on an Illumina MiSeq.

### Sequencing analysis

Raw 16S sequencing data was demultiplexed in Qiita [53] and processed using Deblur [54]. Microbiome data analysis, including feature table filtering, rarefaction, alpha diversity, beta diversity, and taxonomic assignments, was performed with QIIME 2 [55] v 2017.10. Taxonomic assignments used the naive bayes sklearn classifier in QIIME 2 trained on the 515F/806R region of Greengenes 13_8 99% OTUs. As many of the samples were low biomass, DNA contaminants from sequencing reagents and kits had a substantial impact on the dataset. Negative controls that went through the entire pipeline, from DNA extraction to sequencing, were used to catalog these contaminants. Sequences that appeared in negative controls were removed from the lavage samples, excluding a sample-abundant *Lactobacillus* sequence that was believed to be well to well contamination. Mitochondria and chloroplast 16S sequences were also removed.

Community state types were assigned using the hierarchical clustering with Ward’s linkage (SciPy) of Euclidean distances calculated on a table rarefied to 1500 sequences per sample and validity of clusters assessed with Silhouette Coefficient (sklearn). Data visualizations were generated with the python packages seaborn [56], matplotlib [57], and EMPeror [58] was used to create PCoA plots.

Statistical analysis was performed using SciPy [59] to perform the Mann-Whitney U for comparing pathogen CFU between mCSTs and Bray-Curtis distances between PBS and GBS mice at individual timepoints, and to perform spearman correlations for log ratios and Bray-Curtis distance. PERMANOVA implemented in QIIME 2 was used to assess statistical significance of beta diversity clusters.

## Additional files

Additional file 1: Community States are Consistent between Datasets. Heatmap displaying mCST and sequencing data from both studies. (PDF 570 kb)

Additional file 2: Rarefaction Curves for GBS Challenge Experiment. Observed OTU Rarefaction curves. (PDF 588 kb)

Additional file 3: GBS CFU is only recovered in mCST VI mice on Day 21. Displays GBS CFU in tissues by mCST for days 14 and 21. (PDF 280 kb)

Additional file 4: Murine Study Design. Overview of experimental design for both studies. (PDF 372 kb)

## Abbreviations

CFU: Colony forming units
CST: Community state type
GBS: Group B *Streptococcus*
mCST: Murine community state type
OTU: Operational taxonomic units
PBS: Phosphate-buffered saline
