# Supplementary material for: The murine vaginal microbiota and its perturbation by the human pathogen group B Streptococcus
Source: BMC Microbiol. 2018 Nov 26;18:197. doi: 10.1186/s12866-018-1341-2 (PMC6260558; doi:10.1186/s12866-018-1341-2)
Supplement: Supplementary file 1 — Community States are Consistent between Datasets. Heatmap displaying mCST and sequencing data from both studies. (PDF 570 kb) [file 12866_2018_1341_MOESM1_ESM.pdf]

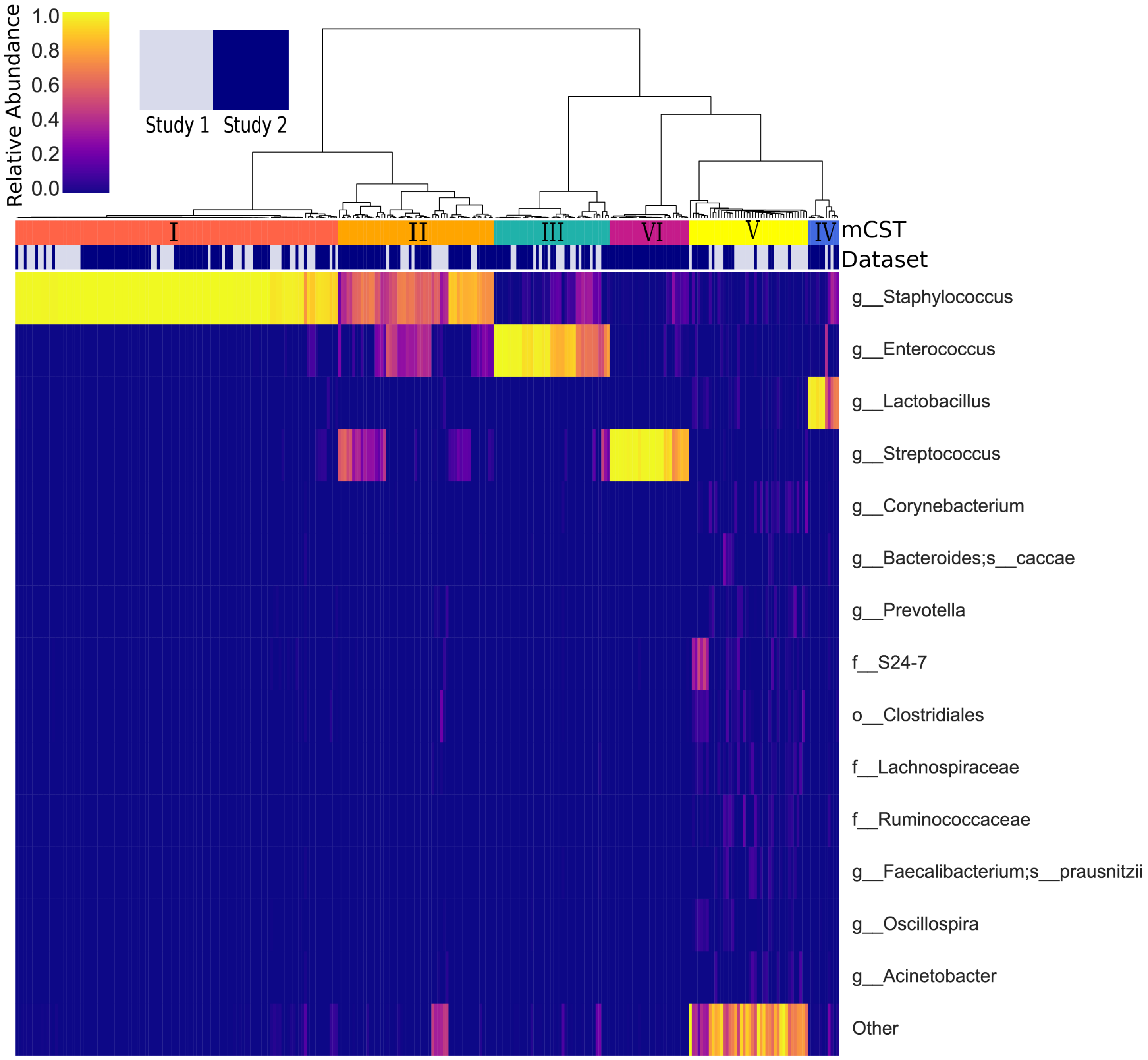

Additional file 1. **Community States are Consistent between Datasets**. Deblurred data from the staging study and GBS colonization study were merged and rarified to 500 sequences per sample. Merged data was clustered by community state with Ward's linkage of Euclidean distances (silhouette score 0.659, sklearn). Bacterial abundances are indicated by heatmap intensity corresponding to the colorbar ranging from purple to yellow
