# Supplementary material for: The murine vaginal microbiota and its perturbation by the human pathogen group B Streptococcus
Source: BMC Microbiol. 2018 Nov 26;18:197. doi: 10.1186/s12866-018-1341-2 (PMC6260558; doi:10.1186/s12866-018-1341-2)
Supplement: Supplementary file 2 — Rarefaction Curves for GBS Challenge Experiment. Observed OTU Rarefaction curves. (PDF 588 kb) [file 12866_2018_1341_MOESM2_ESM.pdf]

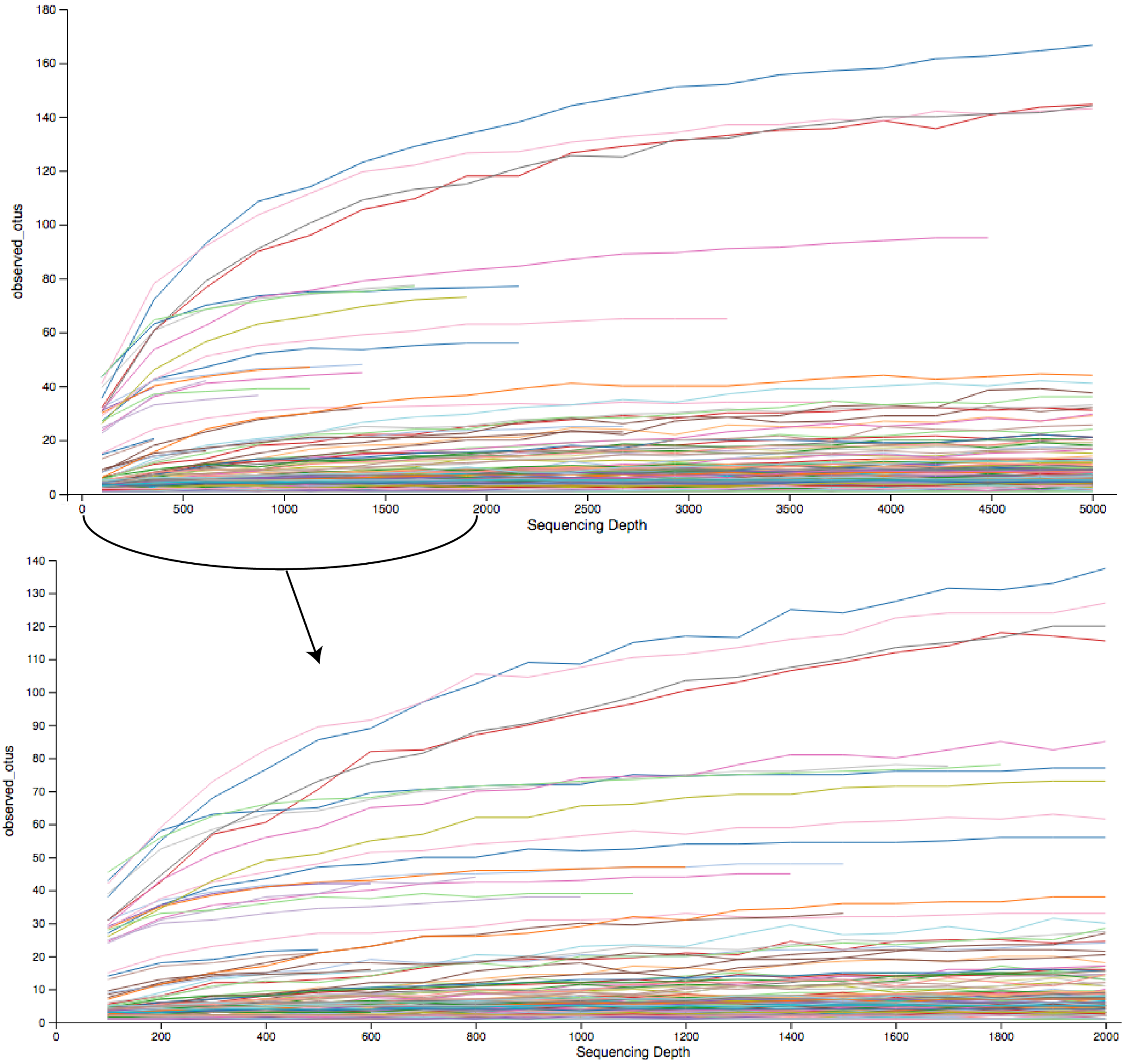

Additional file 2. **Rarefaction Curves for Estrous Staging Experiment**. Curves were generated with QIIME2 to calculate Observed OTUs alpha diversity for samples with contaminant, mitochondria, and chloroplast reads removed. Samples with less than 500 reads are not depicted in these images.
