# Supplementary material for: The murine vaginal microbiota and its perturbation by the human pathogen group B Streptococcus
Source: BMC Microbiol. 2018 Nov 26;18:197. doi: 10.1186/s12866-018-1341-2 (PMC6260558; doi:10.1186/s12866-018-1341-2)
Supplement: Supplementary file 3 — GBS CFU is only recovered in mCST VI mice on Day 21. Displays GBS CFU in tissues by mCST for days 14 and 21. (PDF 280 kb) [file 12866_2018_1341_MOESM3_ESM.pdf]

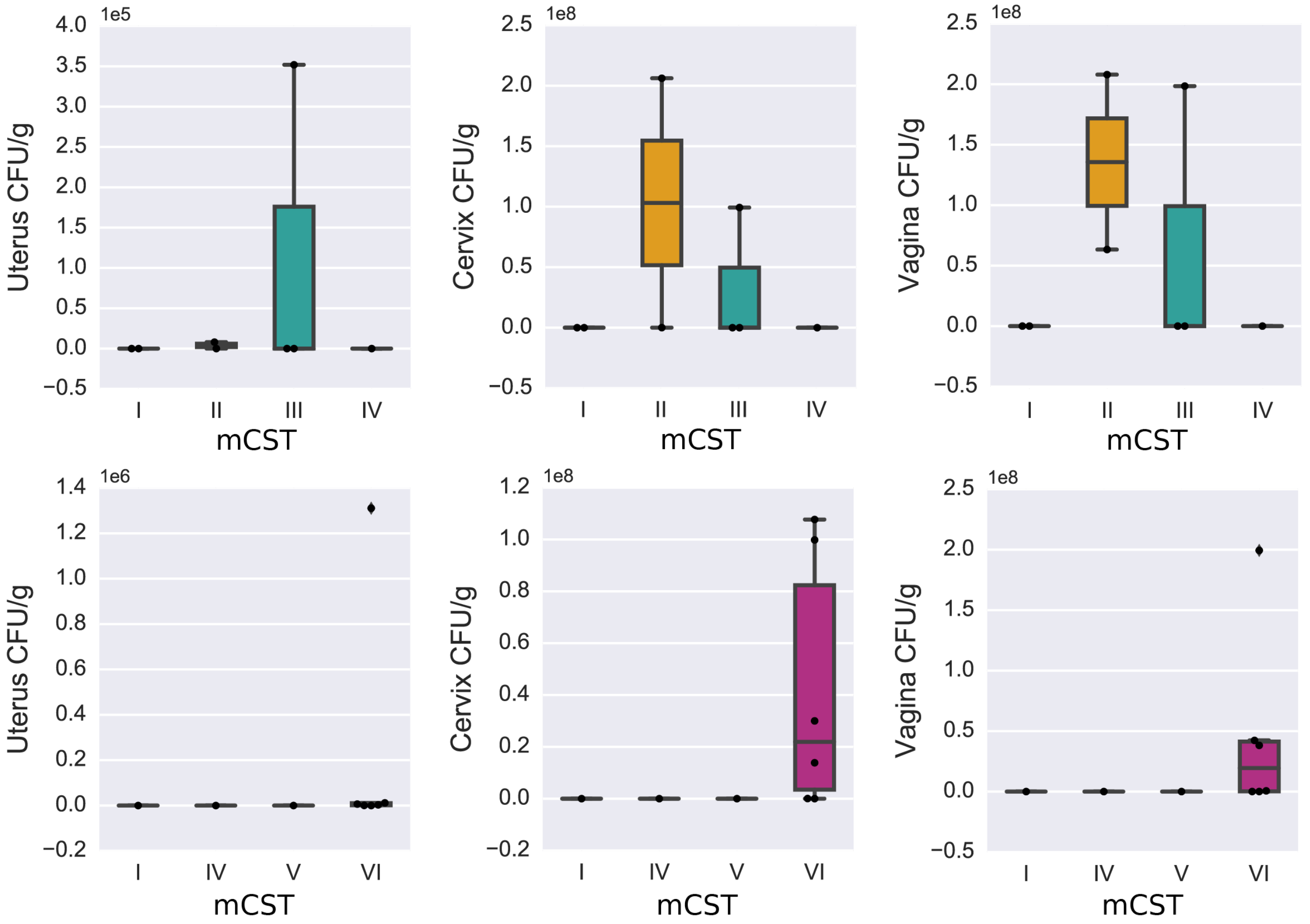

Additional file 3. **GBS CFU is only recovered in mCST VI mice on Day 21**. Tissue CFU/g on days 14 (top) and 21 (bottom) grouped according to mCST classification.
