# Supplementary material for: The murine vaginal microbiota and its perturbation by the human pathogen group B Streptococcus
Source: BMC Microbiol. 2018 Nov 26;18:197. doi: 10.1186/s12866-018-1341-2 (PMC6260558; doi:10.1186/s12866-018-1341-2)
Supplement: Supplementary file 4 — Murine Study Design. Overview of experimental design for both studies. (PDF 372 kb) [file 12866_2018_1341_MOESM4_ESM.pdf]

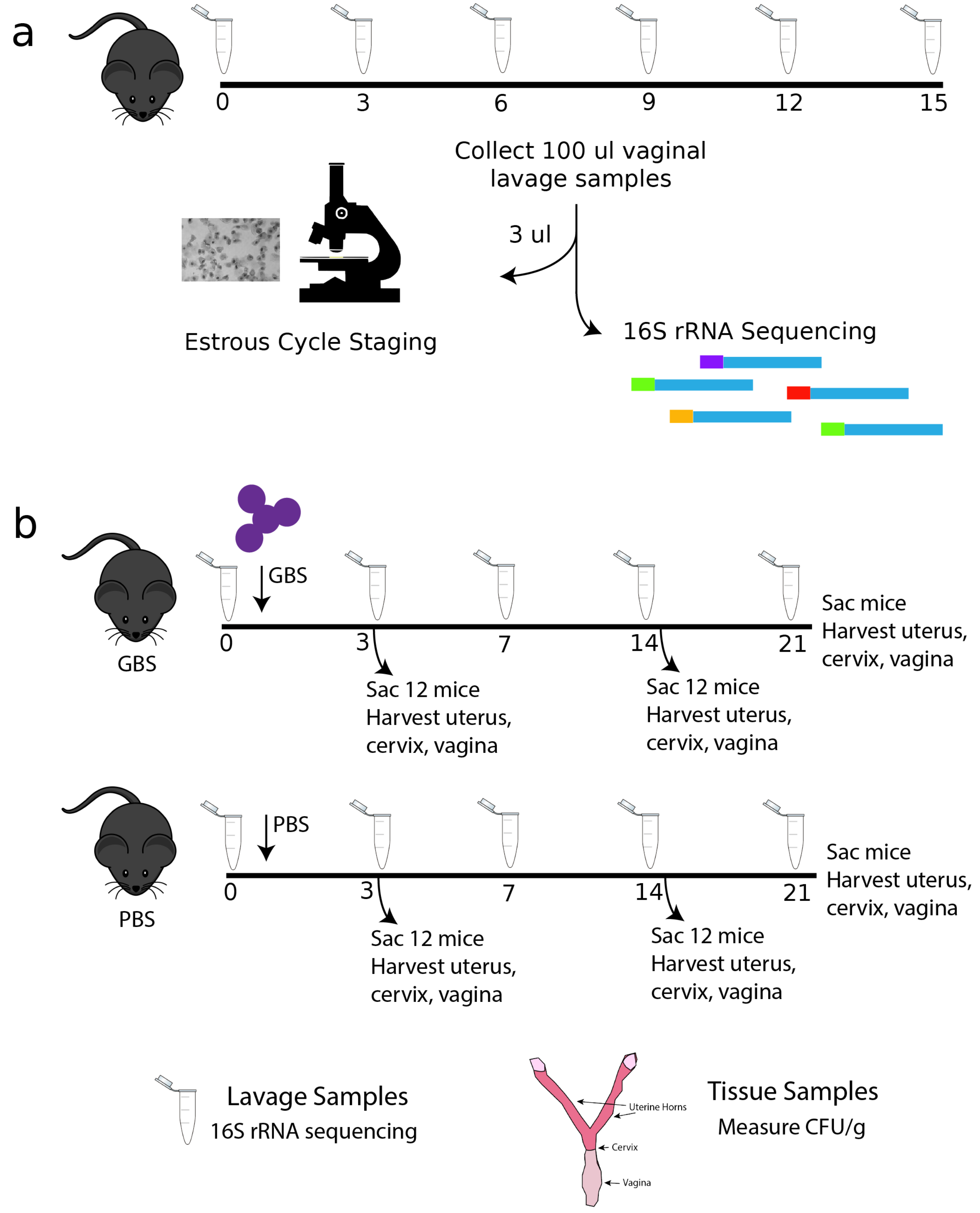

Additional file 4. **Murine Study Design** (**a**) Study 1: Estrous Cycle staging experiment, (**b**) Study 2: GBS Pathogen Challenge experiment. Mouse image is an open source image taken from Pixabay.
